# An Apology

**Published:** 1872-01

**Authors:** 


### AN APOLOGY.

In consequence of the publication of the Index with this number of the BISTOURY, we have been compelled to omit most of our editorial matter. The prospectus, premium list, and report of subscriptions received have for the same reason, been dislodged from their usual position—upon inside of cover—to that of facing the title page. This was rendered necessary in the "make up" in order to have the title page and index come in a suitable position for binding.

But we are inclined to believe our readers will pardon these little irregularites when they come to see the evident pains taken in the arrangement of the index, so that they might be in possession of a volume, the contents of which is made so readily accessible. We point with pride and satisfaction to our table of contents, and think our patrons will agree with us that we have furnished them with a vast amount of invaluable information for an exceedingly small sum of money.

